# The Control of Gastrointestinal Parasites of Village Chickens in Africa Using Ethnoveterinary Intervention: A Systematic Review

**DOI:** 10.3390/vetsci12050407

**Published:** 2025-04-26

**Authors:** Dikeledi Petunia Malatji, Tondani Madeleine Ramantswana, Maphuti Betty Ledwaba

**Affiliations:** 1 Department of Agriculture and Animal Health, College of Agriculture and Environmental Science, University of South Africa, Roodepoort 1709, South Africa; 2 Agricultural Research Council, Biotechnology Platform, Onderstepoort, Pretoria 0110, South Africa

**Keywords:** antiparasitic remedies, control measures, helminths, parasitic infections, poultry, smallholder farmers

## Abstract

Gastrointestinal parasites have been reported to have negative effects on chickens reared in rural areas in African countries. Hence, smallholder farmers use ethnoveterinary remedies to control these parasites in village chickens. This study used online databases (ScienceDirect, PubMed, and Google Scholar) to search, gather, and review articles published between January 1990 and June 2024 where ethnoveterinary intervention was considered to control gastrointestinal parasites, following PRISMA guidelines. A total of 540 articles were generated, and only 17 articles passed the screening process and were considered for further analysis. The findings of this review indicate that ethnoveterinary remedies are used by smallholder farmers to control gastrointestinal parasites such as *Ascaridia galli*, *Capillaria* spp., *Choanotaenia infundibulum*, *Eimeria* spp., *Heterakis gallinarum*, and *Raillietina cesticillus* in village chickens. *Aloe* spp., *Carica papaya*, *Musa paradisiaca*, and *Venonia amygdalina* were reported as the most-preferred medicines, and farmers utilized different plant parts ranging from leaves (48.8%), bark and roots (12.2%), tubers (7.3%), and seeds (4.9%). These remedies are administered per os, topically, and dermally. The current review successfully summarized ethnoveterinary intervention used by smallholder farmers to control gastrointestinal parasites in chickens found in African countries. Therefore, there is a need to investigate the efficacy of these identified ethnoveterinary medicines against gastrointestinal parasites in chickens.

## Simple Summary

In African rural communities, village chickens are essential for livelihood support, poverty alleviation, and food security. They are exposed to gastrointestinal parasites as a result of traditional farming methods. This review sought to document and analyze the indigenous veterinary interventions used by smallholder farmers in Africa to manage gastrointestinal parasite infections in village chickens. This review reveals that ethnoveterinary remedies such as *Aloe* spp., *Carica papaya*, *Musa paradisiaca*, and *Venonia amygdalina* are a common approach for small-scale farmers to manage gastrointestinal parasites. Leaves (48.8%) are the most widely used plant part among farmers, followed by bark and roots, tubers, and seeds and the preferred method of administrating these remedies is orally. Despite their widespread use, there is a lack of research on the effectiveness of these remedies in Africa, highlighting the need for further studies.

## 1. Introduction

Village chickens play a vital role in the livelihood of farmers and their dependents in rural households and landless communities across African countries through poverty alleviation and food security [1,2,3]. However, these chickens are exposed to gastrointestinal parasites due to the nature of production systems practiced in these communities. These parasites result in high mortality rates due to contaminated water sources, feeds, and waste systems in the environment, which exposes chickens to intermediate hosts such as ants, earthworms, grasshoppers, and beetles that assist in spreading parasites [4]. These organisms can withstand harsh environmental conditions and furthermore serve as food for village chickens, making transmission of the infective stage of the parasite to chickens highly possible.

The control of these parasites is compromised by the absence of biosecurity measures and the limited resources for chicken management to avoid the transmission of parasites among individual chickens. However, there are several approaches that are being used to control gastrointestinal parasites, with anthelmintic chemicals being one of them. This control method is relatively costly, and the majority of smallholder farmers are not able to expend their meager income to buy them [5]. Furthermore, it is characterized by a widespread occurrence of drug resistance [6] and residual products that contaminate animal products. As a result, smallholder farmers resort to using ethnoveterinary remedies to control these parasites in village chickens [7,8,9]).

A study by Bizimana [10] reported that only a fraction of the information on ethnoveterinary medicines has been uncovered and documented because some farmers regard it as a family secret, while others view it as old-fashioned. However, different types of medicinal remedies have been reported to be used by smallholder farmers to control gastrointestinal parasites in livestock [7,8,9]. Furthermore, they use different forms of plants such as trees, herbs, and shrubs, and the plant parts that are used include seeds, roots, barks, flowers, bulbs, fibers, peels, stems, latex, and leaves [11].

However, these medicines are characterized by a lack of standardization of raw materials, non-existence of criteria for quality control, no standardized processing methods and dosage formulation, and their efficacy is not well known [12]. The main aim of this review was to explore the ethnoveterinary interventions practiced by smallholder farmers against gastrointestinal parasites in village chickens in Africa. Furthermore, this review highlights the plant parts and route used by stallholder farmers in Africa to administer ethnoveterinary remedies.

## 2. Materials and Methods

This review was performed in accordance with the Preferred Reporting Items for Systematic Reviews and Meta-Analyses (PRISMA) guidelines. A concept note was generated prior to the start of the review and served as the protocol throughout the process.

### 2.1. Literature Search Strategy

A total of three databases were used to conduct a systematic search of literature targeting articles published from January 1990 to June 2024. D.P.M., B.M.L., and T.M.R. searched the Science Direct, PubMed, and Google Scholar databases, respectively, using these keywords: “ethnoveterinary intervention”, “indigenous chickens”, “gastrointestinal parasites”, “Africa”, and “control measures”, following PRISMA guidelines [13]. During the publication search, keywords were used individually and the Boolean operators “NOT”, “AND”, and “OR” were used to combine keywords.

### 2.2. Inclusion and Exclusion Criteria

All of the articles used in this study were screened and considered eligible if they met these criteria: (i) the study needed to be conducted in Africa, (ii) the articles were original research published between January 1990 and June 2024, (iii) gastrointestinal parasites of indigenous chickens were investigated, and (iv) the work reported on ethnoveterinary interventions used to control gastrointestinal parasites in chickens. Experimental studies, case studies, reviews, books, theses, and articles not written in English; that did not report on gastrointestinal parasites in indigenous chickens; that did not contribute towards answering the research questions; and that were published outside the 1990–2024 year limitation were excluded.

## 3. Results

### 3.1. Database Search Outcome

**Figure 1:**
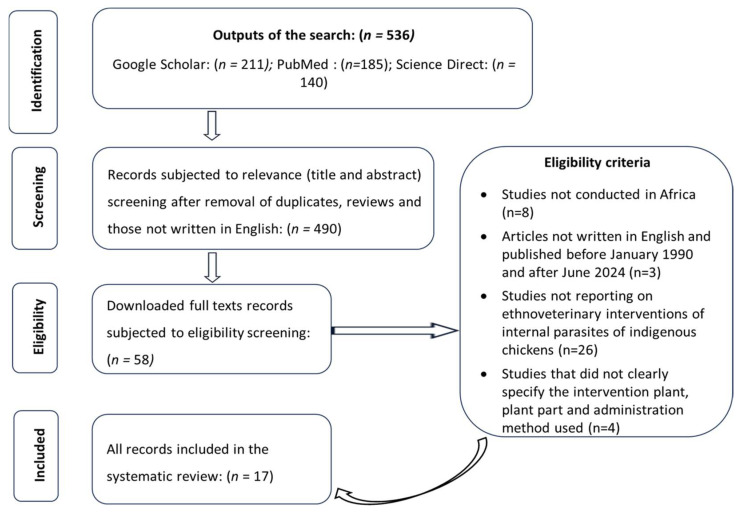
An outline of the database search and screening processes, following the PRISMA guidelines.

The search and pre-screening within the databases generated a total of 536 (Google Scholar = 211, PubMed = 185, and Science Direct = 140) articles (Figure 1). A total of 24 duplicate and 22 review articles were excluded before screening the titles and abstracts, which resulted in the exclusion of 436 additional articles that were not relevant for review. The full text of the remaining records (*n* = 58) was downloaded and screened for eligibility; hence, 41 articles were excluded and only 17 were deemed eligible for inclusion and further consideration in this review.

### 3.2. Data Extracted from the Included Studies

The included studies were from eight African countries, with most of them conducted in Ethiopia (*n* = 5), South Africa (*n* = 3), and Zimbabwe (*n* = 3). Moreover, two articles were conducted in Botswana, while only one article per country was included from the Democratic Republic of Congo, Kenya Tanzania, and Zambia (Table 1). The studies employed various research instruments, which included structured questionnaires (SQ), semi-structured questionnaires (SSQ), and focus group discussions (FGD), and most studies used one method to collect the data. However, studies conducted by Muchadeyi et al. [14] and Matekaire and Bwakura [15] used a combination of SQ and FGD, while Tomeka et al. [16] used SQ and SSQ together. In the case of parasites, most studies indicated endo/internal parasites, gastrointestinal parasitic infections, or worms, while other studies specified parasite names, which included *Ascaridia galli*, *Capillaria* spp., *Choanotaenia infundibulum*, *Eimeria* spp., *Heterakis gallinarum*, and *Raillietina cesticillus*. These parasites were shown to be controlled through several ethnoveterinary interventions (Table 1), and various plant parts such as bark, bulbs, fruit, leaves, roots, seeds, stems, tubers, and/or whole plants were used and administered —either orally or topically. Bark, leaves, and roots were the most-used plant parts, while stems and whole plants were the least recorded.

### 3.3. Gastrointestinal Parasite Control Methods

**Figure 2:**
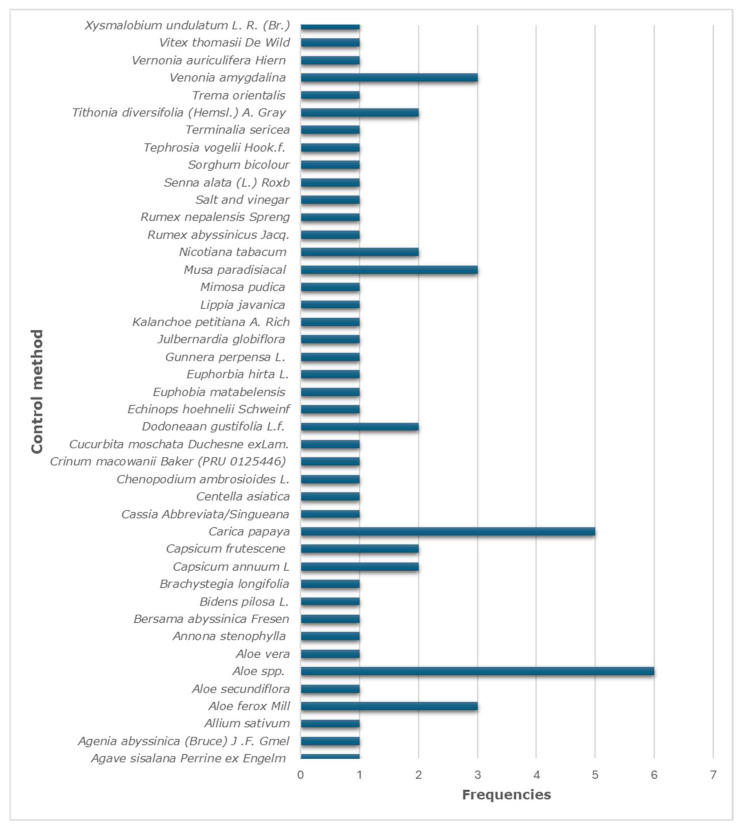
Ethnoveterinary medicines/plants associated with the control of gastrointestinal parasites in chickens, highlighting the most cited plants and their frequency across the reviewed studies, with *Carica papaya* and *Aloe* spp. emerging as the most commonly used.

A total of 43 ethnoveterinary medicines were reported among the included articles (Figure 2). *Aloe* spp., *Musa paradisiaca*, *Carica papaya*, *Aloe ferox* Mill, and *Venonia amygdalina* from the botanical families of Asphodelaceae, Musaceae, Caricaceae, Asphodelaceae, and Asteracea, respectively, were the most medicines/plants associated with the control of chicken gastrointestinal parasite. A total of 9.5% of medicines were recorded in two studies, while the majority of ethnoveterinary medicines (78.5%) were reported in a single study each.

### 3.4. Plant Parts Utilized to Control Gastrointestinal Parasites in Village Chickens

**Figure 3:**
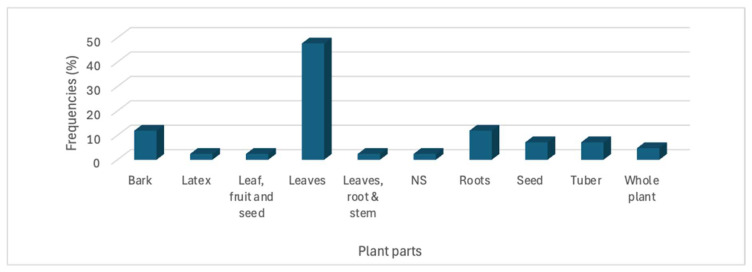
Proportion of plant parts used for treatment of gastrointestinal parasites in chickens. The figure illustrates the frequency of use of various plant parts, with leaves being the most prevalent, followed by bark, roots, tubers and seeds.

The reviewed articles showed that the majority of smallholder farmers predominantly used leaves (47.62%, 20/42), followed by bark and roots at 11.9% (5/42) each, tubers (7.1%, 3/42), and seeds and whole plants at 4.76% (2/42) each as traditional remedies (Figure 3). Remedies involving the whole plant (e.g, leaves, roots, stems, flowers, roots, and seeds); mixtures of leaves, fruit, and seeds; or combinations of leaves, roots, stems; and/or those where plant parts were not specified (NS) were less used, reflecting a lower preference or availability among smallholder farmers in Ethiopia.

The current review indicates that smallholder farmers across various African regions use different methods for administering ethnoveterinary remedies to control gastrointestinal parasites in village chickens. A substantial number of farmers from the reviewed studies prefer oral administration, which accounts for 70.6%. This was followed by topical applications to the skin or mucous membranes (11.8%) and routes that were not specified (11.8%). The simultaneous use of both oral and dermal methods was also reported but is the least preferred approach overall.

## 4. Discussion

Village chickens play a vital role in the livelihood of smallholder and backyard farmers, as well as in the broader community across African countries [25,28,29], as they provide animal protein, generate income, and fulfil various socio-cultural roles [25,28]. However, these chickens can become infected by gastrointestinal parasites, which severely impact their health and hinder advancements in smallholder poultry production and overall sector performance [29]. Smallholder farmers, especially in rural areas, often face challenges accessing veterinary services due to their limited availability and high cost [30]. Conventional Western drugs often fall short in managing these parasites, leading to farmers frequently relying on ethnoveterinary medicine [25]. The constant use of these synthetic remedies can be toxic to chickens [31] and/or cause resistance in the infecting strains [32]. Moreover, products from continuously treated chicken can have harmful implications on human health too [33]. Hence, Jamil et al. [30] indicated that there should be an increase in awareness of the beneficial antiparasitic capabilities of ethnoveterinary medicines.

The current review appraised 17 articles reporting on ethnoveterinary interventions against gastrointestinal parasites in village chickens in Africa. A lot of work on the use of ethnoveterinary remedies in Africa has previously been conducted; however, most research targeted ectoparasites [34,35,36], diseases in livestock in general [20,37], and human health conditions [38,39]; this is the reason for the limited number of articles included in this review. In addition, the studies that reported gastrointestinal parasites in chickens mostly targeted broilers, not indigenous chickens.

This study showed that a reasonable number of ethnoveterinary plants are used by smallholder farmers to control gastrointestinal parasites in village chickens. This might be due to the fact that most farmers, particularly resource-poor farmers in remote rural areas, have minimal access to veterinary services and medicinal drugs available on the market to control the diseases and parasites they may encounter [30]. The most reported plants include *Aloe* spp., *Musa paradisiaca*, *Carica papaya*, and *Vernonia amygdalina*. The beneficial uses of *Aloe* spp. have also been reported previously [40,41] and it has been shown to help in the treatment against multiple health conditions in livestock, including chickens. *Aloe* spp. is prevalent in the Eastern and Southern countries of Africa as it occurs in dry grassland regions [42]. The need to assess the best dosage for an efficient efficacy of *Aloe* spp. is necessary as it is evident with the number of reports that it is used to treat a wide range of health conditions.

*Carica papaya* is a very palatable perennial fruit, and the whole plant parts such as the bark, fruit, leaves, peels, pulp, seeds, and roots are known to have pharmacological uses to treat diseases. In humans, it has been reported to be used for the treatment of high blood pressure, digestive disorders, cancers, intestinal worms, colic, stomach cramps, and/or dyspepsia [43,44]. In animals, specifically chickens, it has been reported to be used for the treatment of *Ascaridia galli* parasites [45]. It has also been reported to be effective at promoting growth in chickens and for the control of coccidiosis [46] and internal parasites in general [18,19]. Previous studies [21,47] have also shown that *Vernonia amygdalina* is effective at treating these health conditions in chhicken. Other plants such as *Musa paradisiaca* [48] and *Aloe* spp. [17] have also been shown to be effective against *Eimeria* spp., which is responsible for coccidiosis.

The reviews also showed that the smallholder farmers preferred using plants and herbs because of their bioactive compounds, which include antioxidant, antimicrobial, antiparasitic, anti-diabetic, and anticancer effects, among others [30,49]. These properties are beneficial for maintaining health and generally have minimal adverse effects [30]. Plant preparations often included extracts from various parts of the plant, such as fruit, seeds, leaves, bark, stems, and roots [30]. Other studies [50,51] showed that farmers in different regions of Ethiopia indicated an overall of 45.8% preference for the use of leaves when preparing plant remedies; hence, this review also showed that leaves are the most commonly used plant parts for preparing remedies for chicken diseases (48.8%). Nguessan et al. [52] also indicated that the leaves of *Annona senegalensis* appeared to be more effective in the control of Strongyles and presumably Coccidia species in small ruminants, as compared with other parts of this plant. In addition, studies reviewed by Jamil et al. [30] indicated that onion, garlic, and mint are commonly used to treat parasitic gastrointestinal infections in animals and birds. Additionally, Suntebo [53] recorded that farmers in the Gamo Zone, Southern Nations, Nationalities, and Peoples Region of Ethiopia commonly use ingredients such as ash, salt, garlic, lemon, butter, oil (fat), ginger, local plants, pepper, antibiotics like tetracycline, and Aloe Vera juice for treating diseases. These ingredients are mixed with water and administered to sick chickens [53].

This study shows that a high proportion (70.6%) of smallholder farmers prefer oral administration of remedies. The studies by Lulekal et al. [54] and Wodegebriel et al. [50] also corroborate that most medicinal plants are administered orally (82.6%). This is not surprising as some smallholder farmers do not have experience administering medicine via intravenous, intramuscular, and subcutaneous routes; hence, their preferred route is oral administration. Moreover, the oral route is preferred because of its ease of administration and many chickens can be medicated at the same time [55]. The route of administration is known to control the rate and extent of absorption of medicine; therefore, it is advisable to use the correct administration to avoid suboptimal medicinal effects [56]. It has previously been indicated that the effectiveness of ethnoveterinary plant extracts in the control of gastrointestinal parasites is mainly influenced by aspects such as the infecting parasite and its life stage, the part of the plant that was used, the route of administration, and the extract dose provided [57].

Previous studies have indicated that the increased awareness and the desire to use plant remedies resulted from drug resistance incidents occurring when using the synthetic drugs [58], as well as the accessibility and low toxicity of these ethnoveterinary plants [59]. Even though Al-Fifi [60] showed a lower survival rate for indigenous chickens with coccidiosis treated with leaf extracts of *Azadiratcha indica*, *Carica papaya*, and *Vernonia amigdalina* compared with those treated with coccidiostatic, the survival rate was still significantly higher than that of the control group.

The articles reviewed in this study had notable limitations. Specifically, some did not report the specific parasite species they targeted, the particular plant parts used, or the route of administration, which are crucial details for understanding ethnoveterinary interventions to treat parasites in chickens. This lack of information highlights the need for more detailed and rigorous study in this research focus area. While East Africa is known for its rich diversity of medicinal plants with antiparasitic properties, the majority of articles used in this study were from Southern Africa. This regional focus can provide valuable insights into the specific plant species and traditional practices used in that area to treat internal parasites. Studies from all African regions could contribute to a better understanding of the use of these plant-based remedies.

## 5. Conclusions

Gastrointestinal parasites such as *Ascaridia galli*, *Capillaria* spp., *Choanotaenia infundibulum*, *Eimeria* spp., *Heterakis gallinarum*, and *Raillietina cesticillus* are a problem in chickens reared in smallholder farming systems. The findings of this review indicate that smallholder farmers in African countries use ethnoveterinary medicines to control gastrointestinal parasites in indigenous chickens. They prefer using the leaves of plants like *Aloe* spp., *Carica papaya*, *Musa paradisiaca*, and *Venonia amygdalina*, which they administer orally. There is a dearth of information on the efficacy of these remedies in Africa; thus, there is a need for future studies to investigate and explore the usefulness of these control strategies against gastrointestinal parasites.

## Figures and Tables

**Table 1:** List and characteristics of 17 eligible articles included in the systematic review.

Country	Data Collection Methods	Parasites	Control Method	Plant Parts	Route of Administration	Reference
Botswana	Structured questionnaire	*Eimeria* spp. (Coccidiosis)	*Aloe* spp. *Aloe ferox* Mill	Leaves	Oral	[17]
Botswana	Structured questionnaire	Internal parasites	Salt and vinegar	NS	Oral	[7]
Congo	Semi-structure interview	Gastrointestinal parasitic infections	*Bidens pilosa* L. *Carica papaya* L. *Chenopodium ambrosioides* L. *Cucurbita moschata* Duchesne exLam. *Euphorbia hirta* L. *Senna alata* (L.) Roxb *Tephrosia vogelii* Hook.f. *Tithonia diversifolia* (Hemsl.l.) A. Gray *Vitex thomasii* De Wild	Leaves Leaves Leaves Seeds Whole plants Roots Leaves Leaves Roots	Oral	[18]
Ethiopia	Structured questionnaire	Internal parasites	*Carica papaya*	Leavesf, fruit, and seeds	Oral	[19]
Ethiopia	Semi-structured questionnaire	Internal parasites	*Bersama abyssinica Fresen* *Vernonia auriculifera Hiern*	Leaves Whole plants	Oral and dermal	[20]
Ethiopia	Semi-structured questionnaire	Internal parasites	*Agenia abyssinica* (Bruce) J.F. Gmel *Capsicum annuum* L. *Rumex nepalensis Spreng* *Vernonia amygdalina* Del *Kalanchoe petitiana* A. Rich	NS	Oral	[21]
Ethiopia	Semi-structured interview	Internal parasites	*Rumex abyssinicus* Jacq. *Echinops hoehnelii Schweinf* *Nicotiana tobacum* L. (DB.9)	Tubers Roots Leaves	Topical Topical Oral	[22]
Ethiopia	Semi-structure interview	Internal Parasite	*Dodoneaan gustifolia* L.f. Shrub	Leaves	Oral	[23]
Kenya	Structured questionnaire	Worms	*Aloe ferox* *Aloe secundiflora*	NS	Oral	[24]
South Africa	Structured questionnaire	Internal parasites	*Aloe ferox* Mill *Agave sisalana* Perrine ex Engelm, *Centella asiatica*, *Xysmalobium undulatum* L. R. (Br.) *Gunnera perpensa* L. *Millettia grandis* (E.Mey.) Skeels	Leaves Leaves Tubers Tuber Leaves Leaves	Oral	[25]
South Africa	Semi-structure interview	*Choanotaenia infundibulum*, *Raillietina cesticillus*	Sorghum bicolour	NS	Oral	[26]
South Africa	Semi-structure interview	*Ascaridia galli*, *Heterakis gallinarum*, *Capillaria* spp., *Choanotaenia infundibulum*, *Raillietina cesticillus* *Eimeria* spp.	*Aloe* spp. *Allium sativum* *Capsicum annuum*	NS	NS	[8]
Tanzania	Structured and semi-structured interview	Endoparasites	*Tithonia diversifolia (Hemsl.)* A. Gray	Leaves	Oral	[16]
Zambia	Focus Group discussions	Worms	*Cassia Abbreviata/Singueana* *Trema orientalis* *Julbernardia globiflora* *Terminalia sericea* *Brachystegia longifolia*	Bark Bark Bark Bark Bark	Oral	[9]
Zimbabwe	Questionnaire and Focus group discussions	Parasites	*Annona stenophylla* *Capsicum frutescene* *Carica papaya* *Euphobia matabelensis* *Lippia javanica*	Roots Leaves Seeds Leaves Leaves/roots/stems	NS	[14]
Zimbabwe	Questionnaire	*Eimeria* spp. (Coccidiosis)	*Aloe* spp.	Leaves	Oral	[27]
Zimbabwe	Survey	Worms	*Aloe* spp. *Venonia amygdalina* *Musa paradisiaca*	Leaves Leaves Roots	Oral	[15]

NS: Not specified.

## Data Availability

The data supporting the conclusions of this research are presented in a table and accompanying figures within this article.
